# *OsPRR37* and *Ghd7* are the major genes for general combining ability of DTH, PH and SPP in rice

**DOI:** 10.1038/srep12803

**Published:** 2015-08-04

**Authors:** Chuan Liu, Gaoyuan Song, Yanhao Zhou, Xuefeng Qu, Zhibin Guo, Zhenwei Liu, Daiming Jiang, Daichang Yang

**Affiliations:** 1 State Key Laboratory of Hybrid Rice and College of Life Sciences, Wuhan University, 430072, China

## Abstract

Artificial selection of high yield crops and better livestock is paramount importance in breeding programs. Selection of elite parents with preferred traits from a phalanx of inbred lines is extremely laborious, time-consuming and highly random. General combining ability (GCA) was proposed and has been widely used for the evaluation of parents in hybrid breeding for more than half a century. However, the genetic and molecular basis of GCA has been largely overlooked. Here, we present two pleotropic QTLs are accounting for GCA of days to heading (DTH), plant height (PH) and spikelet per panicle (SPP) using an F_2_-based NCII design, the BC_3_F_2_ population as well as a set of nearly isogenic lines (NILs) with five testers. Both *GCA1* and *GCA2* were loss-of-function gene in low-GCA parent and gain-of-function gene in high-GCA parent, encoding the putative *Pseudo-Response Regulators, OsPRR37* and *Ghd7,* respectively. Overexpression of *GCA1* in low-GCA parent significantly increases GCA effects in three traits. Our results demonstrate that two *GCA* loci associate with *OsPRR37* and *Ghd7* and reveal that the genes responsible for important agronomic traits could simultaneously account for GCA effects.

One of the most fundamental functions of living organisms is to pass on to their progeny advantageous traits that ensure positive interactions with their environment, whereas any unfit traits are gradually eliminated by natural environmental pressures. This is the well-known evolutionary theory of natural selection^1^. Similarly, artificial selection is a process whereby humans select preferred traits during an organism’s breeding. To meet the increasing global demand for food, artificial selection of high-yield crops and improved livestock has been widely applied and is of paramount importance to hybrid breeding programs. However, the selection of parents from a suite of inbred lines is extremely laborious, time-consuming and random. In addition, parents with excellent agronomic traits do not always pass on those traits to their progeny. Thus, Sprague and Tatum proposed the concept of general combining ability (GCA) to evaluate breeding parents^2^. GCA has been widely and successfully used as a metric for selecting elite parents in conventional livestock and crop breeding programs.

The first research regarding the genetic variance of GCA and specific combining ability (SCA) was reported by Griffing (1956) using a diallelic cross-mating design. Since then, it has become possible to produce precise estimates of GCA and SCA^3^. As GCA variation is derived from the additive effect of traits with high heritability, alleles accounting for this additive effect are believed to easily accumulate in progeny as a result of artificial selection. By contrast, SCA is characterised by non-additive effects, including dominance, over-dominance and epistasis, which are considered difficult to resolve in progeny due to their low heritability and their interactions with the environment^4^. Until now, most studies of combining ability have focused on the identification of promising parents^5^,^6^,^7^,^8^,^9^,^10^. However, the genetic and molecular basis of GCA has been largely overlooked. It is important to uncover the genetic basis of GCA to broaden understanding of the molecular mechanisms underlying GCA and improve the selection of elite varieties in hybrid breeding.

Recently, GCA has been used as one of the most reliable ways to predict the performance of hybrids. Several prediction methodologies based on molecular markers and genomic, transcriptomic and metabolic prediction methods have been developed^11^,^12^,^13^,^14^. A GCA-based model with SCA estimates based on molecular marker data has shown high prediction efficacy^11^. Furthermore, a haplotype blocks-based model for hybrid performance prediction has been developed. A comparison between single AFLP marker- and GCA-based approaches has shown that the hybrid performance prediction based on GCA effects was effective for inter-group hybrids with a predominance of GCA over SCA^12^.

More recent studies have attempted to uncover the genetic basis of GCA by employing a quantitative trait locus (QTL) mapping approach^15^,^16^,^17^. Qu *et al.* identified the genetic loci for combining ability that correspond to agronomic traits through QTL mapping using three testcross populations and a backcross recombination inbred line (BCRIL) in rice. The characteristics of the QTLs for combining ability were found to be similar to those of the QTLs for BCRIL performance^16^. Qi *et al.* identified several genetic loci of GCA and SCA in maize using four testers from different heterotic groups and introgression lines (ILs)^17^. Their studies revealed that GCA is a quantitative trait and is controlled by genes that are similar to those of most mapped QTLs. Moreover, our recent studies revealed that the phenotypic predisposition of the parent in F1 hybrid is correlated with transcriptome preference of the positive GCA parent^18^. However, the genes responsible for GCA remain to be uncovered.

In the present work, we identified 13 QTLs that account for the GCA of three agronomically important traits associated with rice grain yield by using an F2-based NCII design with five testers. We also finely mapped two major pleotropic QTLs—*GCA1* and *GCA2*—through the BC_3_F_2_ population and a set of nearly isogenic lines (NILs). We revealed that *GCA1* and *GCA2* encode the putative *Pseudo-Response Regulator OsPRR37* and *Ghd7,* respectively. Furthermore, we confirmed that *GCA1* functions as a positive regulator not only of the agronomic traits *per se* but also of GCA by virtue of the overexpression of *GCA1* (*GCA1*^OX^) in rice. Additional experiments revealed that GCA1 is located in the nucleus and that the loss of the 234 amino acids at its C terminus does not affect its nuclear localisation. Expression profiling of *GCA1* indicated that it was constitutively expressed in all developmental stages and tissues and was highly expressed in leaves, young panicles and stem. Our results revealed that the genes for important agronomic traits could simultaneously account for the GCA effects. These findings provide new insight into the molecular genetic basis of GCA and the relationship between important agronomic traits and the effects of GCA.

## Results

### Variance analysis of combining ability for F_2_ population

To evaluate the GCA effects of DTH, PH and SPP, we used a diallelic cross of six elite rice varieties. We found that Guangluai #4 (GL) exhibited the lowest GCA effects, whereas Teqing (TQ) exhibited the preferred positive GCA effects for three agronomically important traits (see Supplementary Table S1 online). Therefore, GL and TQ were chosen to generate an F_2_ population for genetic mapping. To identify the effects of GCA and SCA in the F_2_ population, variance analysis of GCA and SCA for the three agronomic traits DTH, PH and SPP was carried out on 139 individual plants in the F_2_ population. The mean squares for the GCA effects from the F_2_ individuals and testers as well as the SCA effects from progeny of F_2_ individuals and testers (Testers×F_2_) were found to be significant for all the three agronomic traits (Table 1). The results showed that the variance components of GCA (Vgca) ranged from 68.6%–96.18%, whereas the variance components of SCA (Vsca) ranged from 6.46%–31.4%, indicating that the GCA variance is substantially larger than the SCA variance (Table 1). These results suggested that both additive and non-additive effects contribute to the genetic variation observed among the hybrids and that the additive effect plays a predominant role in controlling the genetic variation of the three traits, whereas non-additive effects play a minor role.

### Identification of QTLs for GCA

Our previous results showed that the GCA effects made a major contribution to the performance of hybrid offspring. To genetically map the GCA loci for the three traits, the GCA values from a segregation population are required as phenotypes for genetic mapping. We first evaluated the GCA effects in 139 individuals derived from the F_2_ population. As shown in Table 2, the GCA effects among 139 individual plants in the F_2_ population ranged from −17.8 to 30.6 for DTH, −10.1 to 9.2 for PH and −22.0 to 15.7 for SPP. These results demonstrated that the values of GCA in the F_2_ population were typical normal distributions, and the mean values of GCA for the three traits were almost zero, suggesting that the GCA effects are quantitative traits. These results indicated that this F_2_ population was suitable for further QTL analysis of GCA.

**Figure 1:**
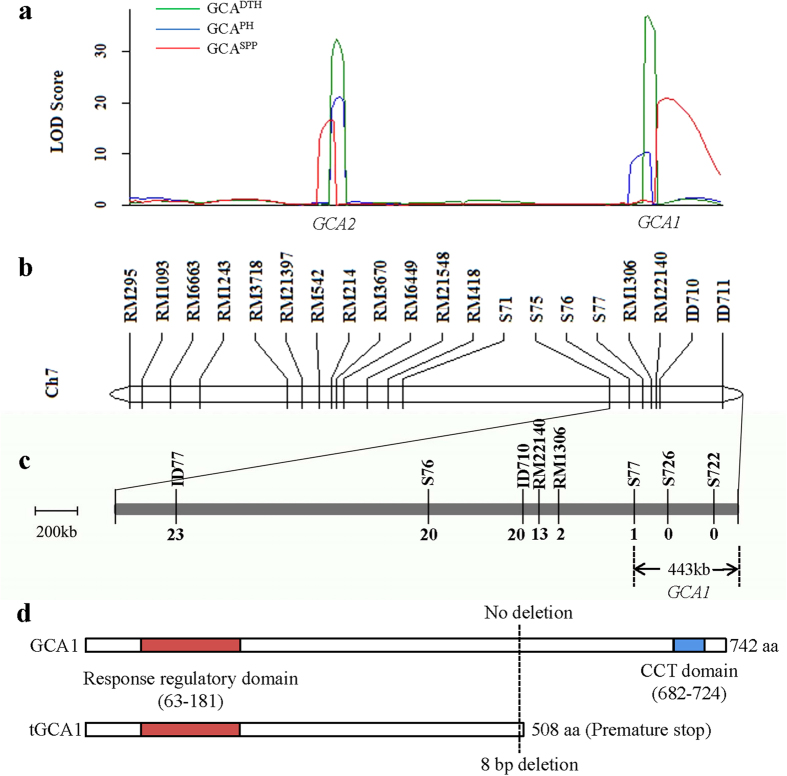
Mapping of the *GCA1* and *GCA2* on chromosome 7 and fine mapping of *GCA1*. (**a**) Two pleotropic GCA QTLs for DTH, PH and SPP were simultaneously detected near the centromere region and the telomere region, respectively, on chromosome 7. (**b**) Linkage map of chromosome 7. (**c**) Fine mapping of *GCA1* with recombinants from BC_3_F_2_-*GCA1* population. The numbers under each molecular marker indicate the number of recombination events detected between *GCA1* and individual markers. (**d**) The protein structure of GCA1 as predicted by PROSITE (http://prosite.expasy.org/)^43^. The structures of full length (GCA1) and truncated (tGCA1) GCA1 protein were presented. The numbers below each domain show the predicted start and end positions. “aa”** **= amino acids.

We then further mapped the QTLs of GCA for the three agronomic traits. A total of 139 individual plants from the F_2_ population and 141 molecular markers (SSRs, InDels and SNPs) were used to construct a linkage map with coverage of approximately 1,763 cM (see Supplementary Fig. S1 online). The GCA values of 139 individual plants from the F_2_ population were obtained from total of 695 TC progenies (see the Method section). A total of 13 major QTLs for GCA of the three agronomic traits were identified on five chromosomes based on a cut-off LOD score ≥ 3.0 (Table 3). Five QTLs for the GCA of DTH were identified on chromosomes 1, 6 and 7, and four of them showed high LOD scores (LOD score > 10). In particular, two QTLs on chromosome 7 explained 29.9% and 34.9% of the variance in the GCA of DTH with additive effects of 6.6 and 7.1 in the F_2_-based TC population, respectively. The LOD peaks of two QTLs were localised at two distant intervals—RM3670-RM6449 and S77-RM1306—indicating that two different loci control the GCA of DTH on chromosome 7. For the GCA of PH, three QTLs were identified on chromosomes 7 and 11. Two QTLs on chromosome 7 explained 42.4% and 16.8% of the GCA variance, respectively. The LOD peaks of the two QTLs for GCA of PH were located within the identical intervals occupied by the other two QTLs for the GCA of DTH, respectively. Furthermore, five QTLs of the GCA for SPP were detected on chromosomes 1, 3, 7 and 11. Surprisingly, we found that two QTLs on chromosome 7 explained 26.3% and 38.2% of the variance in the GCA of SPP, respectively, which coincided with the two QTL intervals of the GCA for DTH and PH. These results suggested that the two clusters of QTLs on chromosome 7 are pleotropic QTLs that simultaneously affect the GCA variation for all the three traits. Therefore, we designated the pleotropic locus on the telomere region as *GCA1* and the other locus on the centromere region as *GCA2* (Fig. 1a,b). Given that *GCA1* and *GCA2* on chromosome 7 explained the highest variance of GCA for three traits, we focused on these two pleotropic QTLs for further study.

### Fine mapping of the QTLs for GCA

To precisely map *GCA1* and *GCA2*, two BC_3_F_2_ populations containing *GCA1* (BC_3_F_2_-*GCA1*) and *GCA2* (BC_3_F_2_-*GCA2*) were developed by continuous backcrossing using GL as the recurrent parent via marker-assisted selection (MAS) with the flanking SSR markers RM3670 and RM1306, respectively (see Supplementary Fig. S2a online). The characterisation of agronomic trait performance indicated that DTH in dominant individuals was increased by 14.2 days in the BC_3_F_2_-*GCA1* population and 12.1 days in the BC_3_F_2_-*GCA2* population compared with the corresponding recessive individuals under natural short day (NSD) conditions. The DTH of dominant individuals from the BC_3_F_3_-*GCA1* and BC_3_F_3_-*GCA2* populations was increased by 23.3 days and 20.9 days compared with those of corresponding recessive individuals under natural long day (NLD) conditions, respectively. Simultaneously, other two traits, PH and SPP, were significantly increased in the dominant individuals of BC_3_F_3_-*GCA1* and BC_3_F_3_-*GCA2* populations (Table 4). Bioinformatics analysis revealed that the *GCA2*-linked marker was located approximately 2 Mb from the pleotropic gene *Ghd7*^19^. Further sequence analysis of *Ghd7* in two parental lines revealed that the null allele type *Ghd7-0* was present in GL, whereas the functional *Ghd7* was present in TQ. Taking together, these results suggested that *Ghd7* is *GCA2*, located near the centromere, and contributed to the GCA variations of DTH, PH and SPP. Moreover, these results showed that *GCA1* was located at the telomere region approximately 20 Mb away from *Ghd7*, suggesting that it may be a new locus. Therefore, we subsequently concentrated on this locus for further fine mapping in the BC_3_F_2_-*GCA1* population.

To obtain sufficient recombinant plants for fine mapping of *GCA1*, we first used the molecular markers encompassing the *GCA1* region to genotype plants in the BC_3_F_2_-*GCA1* population. Two polymorphic SSR markers (RM22140 and RM1306) and two newly developed InDel markers (ID77 and ID710) were used to screen 3,127 individuals from BC_3_F_2_-*GCA1* population. A total of 23 recombinant individuals were identified, and their GCA values for three agronomic traits were obtained by crossing to the tester varieties (see Supplementary Table S2 online). We found that the GCA values of the three agronomic traits from those recombinant individuals were highly correlated based on the linkage analysis. Further genetic analysis of the GCA effects of the 23 recombinants indicated that the *GCA1* interval mapped to the region extending from RM1306 to the end of chromosome, which represents a physical distance of 802 kb. To precisely locate *GCA1* within this region, three SNP markers (S77, S722 and S726) newly developed were used for genotyping within the 802 kb region. Finally, the location of *GCA1* was narrowed to a region of approximately 443 kb extending from S77 to the end of chromosome 7 (Fig. 1c and more details can be found as Supplementary Table S2 online).

### Determining the candidate gene for *GCA1*

To determine the candidate gene responsible for *GCA1*, bioinformatics tools were applied to predict the candidate genes that encompassed the *GCA1* region. A total of 65 predicted genes covered the *GCA1* region, according to the database of the Rice Genome Annotation Project (http://rice.plantbiology.msu.edu/). The expression patterns of those genes in both parents were obtained by RNA sequencing data^20^. Three criteria were used to select candidate genes for further sequencing analysis: 1) the difference of the expression level between high GCA and low GCA parents is >or ≅2.0; 2) the expression level of the gene is high; 3) the known functional importance of predicted protein. Five genes—LOC_Os07g49150 (a putative 26S protease regulatory subunit), LOC_Os07g49230 (a putative ubiquitin-activating enzyme), LOC_Os07g49460 (a response regulator receiver domain containing protein), LOC_Os07g49520 (a putative 2-oxoglutarate dehydrogenase E1 component) and LOC_Os07g49530 (a putative MYB family transcription factor)—satisfied the criteria and were selected for further PCR-based sequencing (see Supplementary Table S3 online). Sequencing results showed that an 8 bp deletion in the tenth exon of LOC_Os07g49460 in GL produced a premature stop codon at the position 1525 bp from the translation start site, whereas no sequence differences were found in ORF regions of the other four genes between GL and TQ. Further studies revealed that LOC_Os07g49460 encodes the known protein OsPRR37, an orthologue of *Arabidopsis Pseudo-Response Regulator 7* (PRR7). The 8 bp deletion in the *OsPRR37* in GL resulted in a 234 amino acid truncation at the C terminus, which led to the loss of the CCT domain, whereas the TQ allele has an entire protein with 742 amino acids (Fig. 1d). Recently, *OsPRR37* was reported to regulate heading date, grain number and plant height as a pleiotropic gene and may contribute to rice cultivation at a wide range of latitudes^21^,^22^,^23^,^24^. Thus, the *OsPRR37* gene was considered as the candidate gene for *GCA1*.

### Confirmation of the gene corresponds to *GCA1*

**Figure 2:**
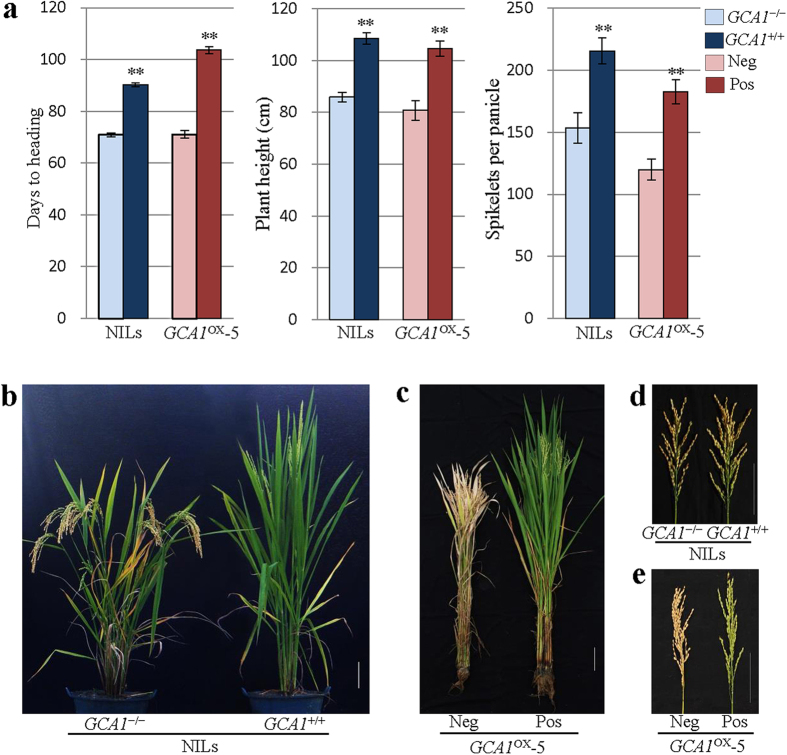
Agronomic trait performance of nearly isogenic lines (NILs) and overexpression line *GCA1*^OX^−5. (**a**) Agronomic traits of different genotypes (*GCA1*^−/−^ or negative (Neg), *GCA1*^+/+^ or positive homozygote (Pos) for NILs and *GCA1*^OX^−5, respectively) under natural long-day (NLD) conditions. Means±standard derivations (SD) were obtained from 20 plants for each genotype (more details can be found as Supplementary Table S4 online). One-tailed *t*-test was used to reveal the significant difference of the agronomic traits between different genotypes of the NILs and *GCA1*^OX^−5. DTH: days to heading; PH: plant heght; SPP: spikelets per panicle; “**” indicates significance at the level of P < 0.01. (**b–e**) Comparisons of plants and panicles of NILs and *GCA1*^OX^−5 with different *GCA1* genotypes. Scale bar = 10 cm.

**Figure 3:**
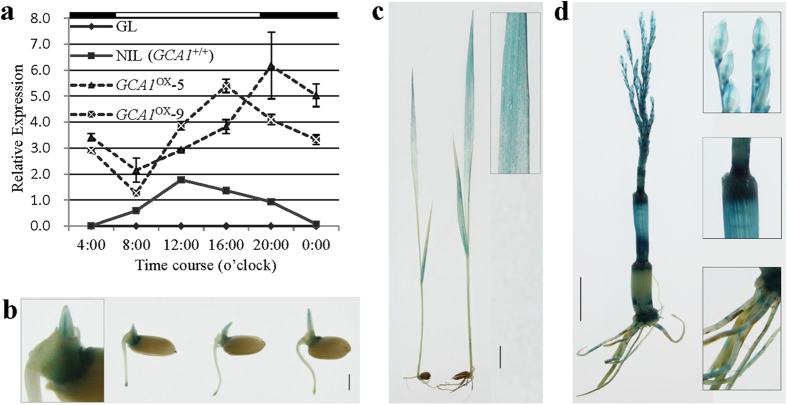
Expression pattern of *GCA1*. (**a**) Diurnal expression of in NIL(*GCA1*^+/+^) and *GCA1*^OX^ lines. The rice Actin1 gene was used as the internal control, and the receptor parent GL was used as the negative control with the TQ-specific qRT-PCR primer. Values are shown as the mean ± SD from three independent experiments. The open and filled bars at top represent the light and dark periods, respectively. The time points below the X-axis show the time course during the day at Wuhan in the summer of 2014. (**b–d**) GUS staining of various organs at different stages in *GCA1* promoter-GUS transgenic plants.(**b**) Germinating seeds, scale bar = 2 mm; (**c**) seedlings at 14 days after germination, scale bar = 1 cm; (**d**) young panicle with intact stem, scale bar = 1 cm.

To verify whether the candidate gene *OsPPR37* is corresponding to *GCA1*, the overexpression transgenic lines (*GCA1*^OX^) carrying 35S:*OsPRR37* were generated. The delayed flowering phenotype was observed in positive transgenic plants. The DTH, PH and SPP of homozygous *GCA1*^OX^−5 plants were increased by 32.5 days, 23.9 cm and 62.6 spikelets, respectively, under natural long day (NLD) condition compared with negative plants (Fig. 2 and more details can be found as Supplementary Table S4 online). We further found that the mRNA level of *OsPRR37* in positive *GCA1*^OX^ lines was significantly higher than that in NIL (*GCA1*^+/+^) (Fig. 3a). The GCA effects of homozygous *GCA1*^OX^ plants in T1 families, NILs (*GCA1*^+/+^ and *GCA1*^−/−^) and NILs (*GCA2*^+/+^ and *GCA2*^−/−^), as well as the parents GL and TQ were evaluated using TC progenies derived from five testers (Table 5). The results indicated that the GCA effects of three independent *GCA1*^OX^ transgenic lines were significantly higher than those of the transgenic recipient parent GL for all the three agronomic traits, suggesting that overexpression of *OsPRR37* significantly increased the GCA effects. Simultaneously, the GCA effects of the three agronomic traits in GL, NIL(*GCA1*^−/−^) and NIL(*GCA2*^−/−^) lines were significantly lower than that in TQ, NIL(*GCA1*^+/+^) and NIL(*GCA2*^+/+^) lines. These results confirmed that *OsPRR37* was the gene corresponding to *GCA1.* Again, the GCA effects of NIL (*GCA2*^−/−^) and NIL (*GCA2*^+/+^) supported that *Ghd7* corresponds to *GCA2*. These results indicated that *GCA1* and *GCA2* significantly increase the GCA of DTH, PH and SPP.

### *GCA1* exhibits a constitutive and diurnal expression pattern

To understand the expression profiles of *GCA1*, the transgenic plants carrying a GUS reporter gene driven by the *GCA1* promoter were generated. The results revealed that *GCA1* was constitutively expressed in all tissues and at all developmental stages (Fig. 3b–d). The most abundant expression of *GCA1* was found in the leaf, young panicle, stem and internode, implying that *GCA1* could function in panicle development and stem elongation, consistent with the GCA effects of DTH, PH and SPP. The expression profile of *GCA1* in NIL (*GCA1*^+/+^) plants exhibited a diurnal pattern (Fig. 3a). The expression level of *GCA1* in NIL (*GCA1*^+/+^) increased from dawn to noon and then gradually decreased until midnight, with a peak expression level at noon under NLD conditions. Surprisingly, the overexpression of *GCA1* driven by the *35S* promoter in *GCA1*^OX^ lines also exhibited a diurnal pattern, but the diurnal expression pattern was altered and peaked around dusk (at 20:00 for *GCA1*^OX^−5 and 16:00 for *GCA1*^OX^−9), with the lowest expression levels observed in the morning (at 8:00 for both *GCA1*^OX^ lines). These results imply that the mRNA level of *GCA1* itself could be regulated by unknown diurnal signal(s).

Previous research has reported that *AtPRR7*, an orthologue of *GCA1*, has a potential bipartite nuclear localisation signal at the C terminus (amino acids 680 to 696). The localisation of the PRR7-GUS fusion was observed to be nuclear in leek epidermal cells^25^. To investigate whether the full-length GCA1 and the truncated GCA1 (tGCA1) had the same nuclear localisation, we monitored the localisation of sGFP-GCA1 and sGFP-tGCA1 via a rice protoplast transient assay. The results showed that both sGFP-GCA1 and sGFP-tGCA1 localised in the nucleus, whereas sGFP alone was detected throughout the cytoplasm (see Supplementary Fig. S3 online). These results showed that GCA1 protein functions in the nucleus, but the truncation of the 234 amino acids at the C terminus does not disturb its nuclear localisation.

## Discussion

In this study, we identified 13 QTLs for the GCA of three important agronomic traits that were mainly associated with grain yield in rice. We identified two major pleotropic QTLs—*GCA1* and *GCA2*—that corresponded to *OsPRR37* and *Ghd7*, respectively, and functioned as positive regulators of GCA for DTH, PH and SPP and these traits *per se*. Our findings revealed that the genes responsible for GCA effects correspond to the genes controlling important agronomic traits. To take another example in this study, two QTLs for the GCA of DTH were found to be located between RM3431 and RM3438 and between RM584 and RM5815 on chromosome 6. The physical regions represented by these SSR markers are close to the physical positions of the genes *Hd1* and *Hd3a* that control flowering time^26^,^27^, indicating that *Hd1* and *Hd3a* may be responsible for the QTL loci for corresponding GCA effects. These results suggested that QTLs for GCA are directly associated with QTLs for these important agronomic traits.

Previous studies have reported that *GCA1* and/or *GCA2* were widely found in elite rice varieties^19^,^21^,^23^,^24^,^28^. For example, the modern elite parents, Minghui 63, Teqing, Huanghuazhan, Guichao #2, Peiai 64S contain both *GCA1* and *GCA2* while 93-11 contains *GCA2*. It is apparently the result of domestic selection during cross-breeding. This pattern explains that high GCA-effect parents could easily pass on the corresponding traits to their hybrid progenies by simultaneously integrating the GCA controlling genes into the genome of hybrid progenies during cross-breeding program. It is well explained why elite varieties could be easily developed from parents with higher GCA effects. Our findings help remove the mystery of GCA that has existed since 1942, even though this trait has been widely used for crop and livestock improvements.

GCA effects have widely been applied by breeders to evaluate breeding parents. The consensus of opinion is that elite breeding parents possess preferred GCA effects, which are characteristics of wide adaptability, high hereditary capacity and elite agronomic traits. Our data show that *GCA1* and *GCA2* encode *OsPRR37* and *Ghd7*, respectively, which have both been reported to be associated with agronomic traits for high grain yield and adaptability at different latitudes^19^,^21^,^23^,^24^. This finding has further confirmed that GCA-controlling genes in elite breeding parents are associated with wide adaptability, important agronomic traits and the capacity to pass on these traits to their hybrid progenies. Our results elucidate why GCA has been effective as a criterion for evaluating the breeding parents. Together, these results also provide new insight into the molecular genetic basis of GCA and the relationship between important agronomic traits and GCA effects. The updated concept of GCA revealed in our findings will provide a useful tool for evaluating and selecting elite parents for molecular design breeding in the future.

In our study, we found that the GCA effect of SPP in NIL(*GCA1*^+/+^) was lower than that of *GCA1*^OX^ transgenic lines, and the GCA effect of SPP in NIL(*GCA1*^+/+^) and NIL(*GCA2*^+/+^) was even lower than zero (Table 5). According to the processing of GCA effect data, GCA value is a relative estimate and the sum of GCA effects for all varieties or inbred lines evaluated must be equal or nearly zero. Thus, the negative value of GCA effect of SPP in NIL(*GCA1*^+/+^) and NIL(*GCA2*^+/+^) was partly caused by the extremely high GCA effect of SPP in parent TQ. Alternatively, the negative value of the GCA effect of SPP in NIL(*GCA1*^+/+^) does not mean inconsistency between NIL(*GCA1*^+/+^) and *GCA1*^OX^ transgenic lines. As in this respect, the GCA effects of NIL(*GCA1*^+/+^) and *GCA1*^OX^ transgenic lines showed the same trends when comparing to the GCA effects of both NIL(*GCA1*^−/−^) or GL. For the other explanation, the GCA difference could be caused by the different expression pattern of *GCA1* between NIL(*GCA1*^+/+^) and *GCA1*^OX^ transgenic lines (Fig. 3a). If it is case, however, it will be an interesting issue remains to know that how the expression pattern of *GCA1* affect GCA effect in future.

Previous studies have reported that *GCA1* belongs to the PRR family, identified as circadian clock-associated PRR homologues of *Arabidopsis*. Each of the family members contains two domains: the Pseudo-Receiver domain at the N terminus and a CCT domain at the C terminus^29^,^30^,^31^. More recently, studies have revealed that the *GCA1* homologue PRR7, together with PRR5 and PRR9, acts as a transcriptional repressor by binding the promoters of the core clock genes *CIRCADIAN CLOCK ASSOCIATED 1* (*CCA1*) and *LATE ELONGATED HYPOCOTYL* (*LHY*) in *Arabidopsis*^32^,^33^. In our study, the diurnal expression pattern of *GCA1* and the nuclear localisation of the GCA1 protein indicated that GCA1 might have a similar function as a circadian-regulated transcription factor in rice. Interestingly, GCA2 also possesses a CCT domain exhibiting a diurnal expression pattern and nuclear localisation^19^. Our study confirms that elimination of the 234 amino acids at the C terminus, which contains the CCT domain, does not alter the nuclear localisation of GCA1, indicating that the nuclear localisation signal does not reside in the C terminus. Recently, it has been shown that the CCT domain is crucial for interactions between *Arabidopsis* PRR5 and target DNA *in vivo*^34^. Therefore, we hypothesize that the low GCA-effect phenotype can be attributed to the loss of the CCT domain at the C terminus, leading to a loss of capacity for interacting with its target nuclear DNA. Therefore, CCT domain could play critical role on the GCA effects for those traits. *GCA1* and *GCA2* might function as transcription factors of various downstream genes in the nucleus by regulating their diurnal expression patterns or expression levels.

## Methods

### Plant materials and the F_2_ population

Estimation of the GCA effects of elite rice varieties was conducted with a diallelic cross design using the Yuetai B (YB), Zhenshan 97 B (ZS), Aijiaonante (AJ), Guangluai #4 (GL), 93-11(L6) and Teqing (TQ) varieties. An F_2_ population with 139 individuals derived from GL×TQ was used in this study. Five varieties—ZS, AJ, GL, L6 and Aizizhan (AZ)—were used as testers to cross to individual plants from the F_2_ population based on the NCII mating design in Lingshui, Hainan Province, in the spring of 2009. The 139 F_2_ plants were crossed with the five testers to evaluate GCA effects and were planted in Wuhan, Hubei Province, in the summer of 2009. Twenty plants of each testcross (TC) progeny were planted in a 16.5 cm × 20 cm area following a randomised complete block design with three replicates.

### Development of the BC_3_F_2_ population for fine mapping

Individual plants from the F_2_ population containing the QTL region of interest were continually backcrossed to GL. The BC_3_F_1_ plants were selected by marker-assisted selection (MAS), and the GCA effects of the recombinant plants from BC_3_F_2_ were evaluated according to the agronomic traits of the TC progeny (Supplementary Fig. S2a online). The agronomic traits of the individual plants in TC populations were recorded. All TC progeny were planted in the same manner as the TC populations of F_2_ plants.

### Measurement of agronomic traits and statistical analyses

For primary mapping, data for the agronomic traits days to heading (DTH), plant height (PH) and spikelets per panicle (SPP) were collected for between 10 and 15 plants from each plot. For fine mapping, the accurate GCA effects of three traits were measured for five plants of non-segregated lines and for between 10 and 15 plants of segregated lines derived from the TC progeny in each replication. Variance analysis and evaluation of the GCA were carried out as previously described^35^. The GCA effects were calculated with the following formula:


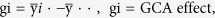


where  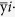 = average of the five TC progenies between individual plant i and the five testers and 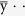 = overall mean of the five TC populations.

The variance analysis and effect evaluation of GCA were processed with DPS software version 7.05^36^. The descriptive statistics—Student’s *t*-test of the agronomic traits—was performed in SPSS statistics software version 17.0 (SPSS Inc., Chicago, IL, USA).

### QTL mapping

A total of 141 polymorphic molecular markers were used to construct the linkage map using plants from the F_2_ population, including 123 SSRs (http://www.gramene.org/), 14 SNPs and four InDel markers developed from previous genome sequencing data for GL and TQ^20^. Conventional polymerase chain reaction (PCR) and polyacrylamide gel electrophoresis (PAGE) techniques were used for SSR and InDel genotyping. For SNP genotyping, the MALDI-TOF MS method was used to identify SNP polymorphisms in the F_2_ population as described previously^37^. Genetic linkage map construction and QTL mapping analysis were conducted with QTL IciMapping software version 3.1^38^. An LOD score of 3.0 was used to indicate the presence of QTLs for each trait. The segregation of the traits for each BC_3_F_3_ family and the corresponding TC progeny was used to distinguish the genotype of the GCA locus in each recombinant plant of BC_3_F_2_.

### Plasmid construction and transformation

The coding sequence (CDS) of the candidate gene *GCA1* was confirmed by PCR-based sequencing of the exons in genomic DNA and cDNA obtained by the mRNA reverse transcription of TQ. The CDS (2,229 bp) and the 3′UTR (623 bp) were artificially synthesised by Gene-Script (Nanjing, China) and were inserted into the binary vector pCAMBIA1300. A CaMV 35S promoter was then fused to the 5′ end of the CDS, resulting in the overexpression construct *pOsPMP625.* To generate the *GCA1* promoter-GUS construct, a 1,884 bp promoter region was amplified by PCR and inserted upstream of the 5′ end of the reporter gene beta-glucuronidase (GUS) in the binary vector pCAMBIA1300, designated *pOsPMP626.* All transgenic plants were generated by introduction into GL through *Agrobacterium*-mediated transformation.

### Evaluation of the GCA effect

NILs corresponding to different genotypes *GCA1* and *GCA2* were obtained from homozygous BC_4_F_2_ plants with more than 90% of the genetic background derived from the GL line, as determined by genome-wide SSR and InDel markers (see Supplementary Fig. S2b,c online). Homozygous NIL (*GCA1*^+/+^ or GCA1^−/−^) and NIL (*GCA2*^+/+^or *GCA2*^−/−^) were determined by using appropriate molecular markers. The homozygous *GCA1*^OX^ plants from the T1 generation were determined by qPCR as described previously^39^. Two NIL sets [NIL (*GCA1*^+/+^ or *GCA1*^−/−^) and NIL (*GCA2*^+/+^ or *GCA2*^−/−^)], three independent homozygous *GCA1*^OX^ lines and the parent lines (GL and TQ) were crossed to five tester varieties [ZS, AJ, L6, YB and Guichao #2 (GC2)] at Lingshui, Hainan Province, in the spring of 2014. The GCA effects and the traits of these lines were evaluated.

### RNA extraction and quantitative RT-PCR

The uppermost fully expanded leaves from at least three individual plants were harvested at 45 days after germination under natural long day (NLD) conditions. Total RNA was extracted from the pooled leaf materials using TRIzol Reagent (Invitrogen, Carlsbad, CA, USA) according to the manufacturer’s instructions and then treated with RNase-free DNase I (New England Biolabs, Hitchin, UK). First-strand cDNA was synthesised using M-MLV reverse transcriptase and oligo (dT) primer (Promega, Madison, WI, USA) as described by the manufacturer. Quantitative RT-PCR was carried out in a total volume of 10 μl, including 2 μl of cDNA template (5- to 10-fold dilutions), 0.5 μl of 10 μM gene-specific primer and 5 μl of SYBR Green qPCR Supermix-UDG with ROX Reference Dye (Invitrogen, Carlsbad, CA, USA). The qRT-PCR was conducted on a StepOne System (ABI) using the following parameters: 95°C for 10 min, followed by 40 cycles of 95 °C for 10 s and 60 °C for 30 s. The relative gene expression levels were calculated according to the ΔΔCT method described previously^40^. The rice *Actin1* gene was used as an internal control to normalise the data.

### GUS staining assay

A GUS staining assay was performed as described in a previous study^41^. Briefly, fresh tissue from the transgenic plants was washed three times with a staining solution containing 50 mM NaPO_4_ buffer (pH 7.0), 2 mM K_3_Fe(CN)_6_, 2 mM K_4_Fe(CN)_6_ and 0.2% Triton X-100 on ice and then incubated with staining solution containing 1 mM X-Gluc at 37 °C overnight. The coloured tissues were photographed with a camera (EOS 500D; Canon).

### Subcellular localisation

The CDS of full-length *GCA1* and truncated *GCA1* (*tGCA1*) were amplified using appropriate primers based on the sequences in TQ and GL, respectively (Supplementary Table 4). Isolated *GCA1* and *tGCA1* were inserted into the *pHBT-sGFP(S65T)* construct at the C terminal of sGFP without a stop codon between sGFP and GCA1 or tGCA1 to create the in-frame fusion constructs sGFP-GCA1 and sGFP-tGCA1, respectively. Rice protoplasts were used for determining subcellular localisation. Protoplast isolation and PEG-mediated transfection were carried out as described in a previous study^42^. The nuclear marker AtH2B fused to a CFP construct (H2B-CFP) driven by *CaMV 35S* promoter was co-transformed with sGFP-GCA1 and sGFP-tGCA1. To avoid fluorescence artefacts that could disturb localisation, an optimised concentration of plasmid DNA (i.e., 15 μg in total) was used for the transient expression assay. Subcellular localisation was visualised on an Olympus Fluoview 1000 laser scanning microscope (http://www.olympus-global.com). Triplicates of each transient expression experiment were performed to obtain a robust result.

## Additional Information

**How to cite this article**: Liu, C. *et al.* *OsPRR37* and *Ghd7* are the major genes for general combining ability of DTH, PH and SPP in rice. *Sci. Rep.* **5**, 12803; doi: 10.1038/srep12803 (2015).

## Supplementary Material

Supplementary Information

## Figures and Tables

**Table 1:** Variance analysis of GCA and SCA of agronomic traits.

Trait	Testers	F_2_	Testers×F_2_	Error	Vgca	Vsca
DTH	243557**	1328**	151**	13	93.5%	6.5%
PH	310630**	324**	108**	18	96.2%	3.8%
SPP	183481**	2391**	959**	227	68.6%	31.4%

^**^Significance at the level of *P *< 0.01.

**Table 2:** Performance of GCA in the F_2_ population.

GCA Trait	GCA range	GCA mean	Skewness	Kurtosis
DTH	−17.8 to 30.6	7.19E-07	0.8	1.1
PH	−10.1 to 9.2	1.44E-06	0.0	−0.2
SPP	−22.0 to 15.7	5.76E-06	−0.3	0.3

**Table 3:** QTL analysis of GCA for three agronomic traits.

GCA Trait	Chr.	Position (cM)	Left marker		Right marker		LOD	PVE (%)	Add	Dom
			Marker name	Position (bp)	Marker name	Position (bp)				
DTH	1	183	S14	22,622,816	S15	24,792,180	3.21	1.71	1.38	−1.49
DTH	6	63	RM3431	8,756,801	RM3438	7,477,690	16.52	10.99	−4.53	−0.80
DTH	6	91	RM584	3,416,638	RM5815	2,928,145	11.21	6.72	−3.30	−1.43
DTH	7	85	RM3670	13,386,382	RM6449	15,357,245	32.41	29.86	6.55	−0.02
DTH	7	213	S77	29,253,591	RM1306	28,894,398	36.90	34.87	7.13	−0.84
PH	7	86	RM3670	13,386,382	RM6449	15,357,245	21.09	42.41	3.70	−0.50
PH	7	213	S77	29,253,591	RM1306	28,894,398	10.20	16.80	2.28	−0.51
PH	11	0	RM6327	364,257	RM6335	479,545	4.27	6.38	−1.44	−0.19
SPP	1	73	RM10043	645,573	RM1	5,050,749	7.42	13.06	3.24	0.80
SPP	3	153	RM416	31,248,603	RM3867	31,540,070	5.27	6.91	2.41	−0.90
SPP	7	83	RM214	12,730,966	RM3670	13,386,382	16.56	26.29	4.57	−0.32
SPP	7	221	ID710	28,724,269	ID711	28,829,798	20.82	38.17	5.51	−1.08
SPP	11	58	RM7463	10,095,283	RM26567	12,830,466	4.58	5.90	2.37	−0.31

The GCA traits (DTH, PH and SPP) used for QTL mapping represent the GCA effect of days to heading, plant height and spikelets per panicle, respectively. Position (cM) indicates the LOD peak position on the genetic linkage map. Position (bp) indicates the physical position of QTL flanking markers on corresponding chromosome (Chr.). PVE (%) represents the percentage of total phenotypic variance explained by the QTL. Add and Dom indicate the additive effect and dominant effect, respectively.

**Table 4:** Trait performance of BC_3_F_2_ plants and BC_3_F_3_ families with recessive (Rec) and dominant (Dom) genotypes under NSD and NLD conditions.

Season	Genotypes	DTH	PH	SPP
		Mean ± SD	Mean±SD	Mean ± SD
2012H	BC_3_F_2_-*GCA1*(Rec)	77.9 ± 1.5	74.0 ± 3.5	154.3 ± 10.1
	BC_3_F_2_-*GCA1*(Dom)	92.1 ± 2.2	80.3 ± 3.0	188.4 ± 13.0
	*P*-value	8.48E-25	3.35E-07	3.03E-11
	BC_3_F_2_-*GCA2*(Rec)	83.0 ± 2.2	71.3 ± 1.9	121.0 ± 14.5
	BC_3_F_2_-*GCA2(*Dom)	95.1 ± 1.6	80.2 ± 2.8	171.9 ± 15.3
	*P*-value	1.98E-21	8.28E-14	7.83E-13
2012W	BC_3_F_3_-*GCA1*(Rec)	63.6 ± 2.5	92.8 ± 3.8	148.4 ± 10.3
	BC_3_F_3_-*GCA1*(Dom)	86.9±2.9	112.4±4.0	194.8±12.6
	*P*-value	3.64E-25	2.25E-17	2.84E-15
	BC_3_F_3_-*GCA2*(Rec)	65.0 ± 1.2	94.9 ± 3.8	136.4 ± 14.9
	BC_3_F_3_-*GCA2*(Dom)	85.9 ± 1.6	108.4 ± 3.0	185.1 ± 13.9
	*P*-value	3.10E-35	5.61E-15	5.64E-13

The mean value±SD (standard deviation) was calculated using 20 plants for each genotype under natural short-day (NSD) conditions in the spring of 2012, Hainan (2012H) and natural long-day (NLD) conditions in the summer of 2012, Wuhan (2012W). The P-value was calculated with One-tailed t-test between the genotypes.

**Table 5:** GCA estimation of NILs and *GCA1* ^OX^ lines with five testers.

Genotype	GCA^DTH^	GCA^PH^	GCA^SPP^
GL	−9.4	−7.6	−10.7
TQ	1.4	10.3	20.6
NIL(*GCA1*^−/−^)	−8.4	−5.9	−8.6
NIL(*GCA1*^+/+^)	9.1	2.8	−1.3
NIL(*GCA2*^−/−^)	−10.1	−8.7	−10.0
NIL(*GCA2*^+/+^)	5.2	3.2	-0.3
*GCA1*^OX^-5	4.4	2.7	2.1
*GCA1*^OX^-6	4.2	0.4	5.7
*GCA1*^OX^-9	3.6	2.9	2.7

Two NIL sets [NIL (*GCA1*^+/+^ or *GCA1*^−/−^) and NIL (*GCA2*^+/+^ or *GCA2*^−/−^)], three *GCA1*^OX^ lines (*GCA1*^OX^-5, *GCA1*^OX^−6 and *GCA1*^OX^−9) and two parental lines (GL and TQ) were used for GCA estimation. GCA^DTH^, GCA^PH^ and GCA^SPP^ represent the corresponding GCA values that were estimated from the whole TC progeny derived from five testers.
