# Supplementary material for: OsPRR37 and Ghd7 are the major genes for general combining ability of DTH, PH and SPP in rice
Source: Sci Rep. 2015 Aug 4;5:12803. doi: 10.1038/srep12803 (PMC4523830; doi:10.1038/srep12803)
Supplement: Supplementary Information [file srep12803-s1.pdf]

***OsPRR37* and *Ghd7* are the major genes for general combining ability of DTH, PH and SPP in rice**

Chuan Liu, Gaoyuan Song, Yanhao Zhou, Xuefeng Qu, Zhibin Guo, Zhenwei Liu, Daiming Jiang, Daichang Yang *

State Key Laboratory of Hybrid Rice and College of Life Sciences, Wuhan University, 430072, China

*Corresponding author: Daichang Yang

Address: State Key Laboratory of Hybrid Rice, College of Life Sciences, Wuhan University, Luojia Hill, Wuhan, Hubei Province 430072, China.

Tel: 86-27-6875-4680

Fax: 86-27-8757-0670

E-mail: dyang@whu.edu.cn

Supplementary Figures

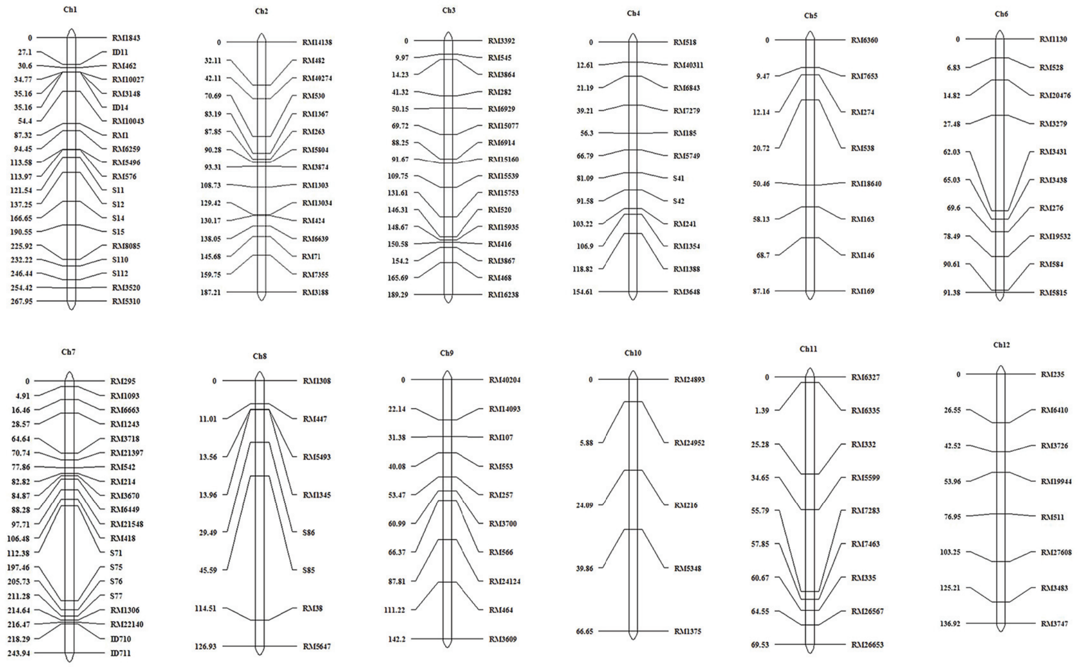

Supplementary Figure S1. Genetic linkage map constructed from 141 molecular markers using 139 plants of the F2 population.

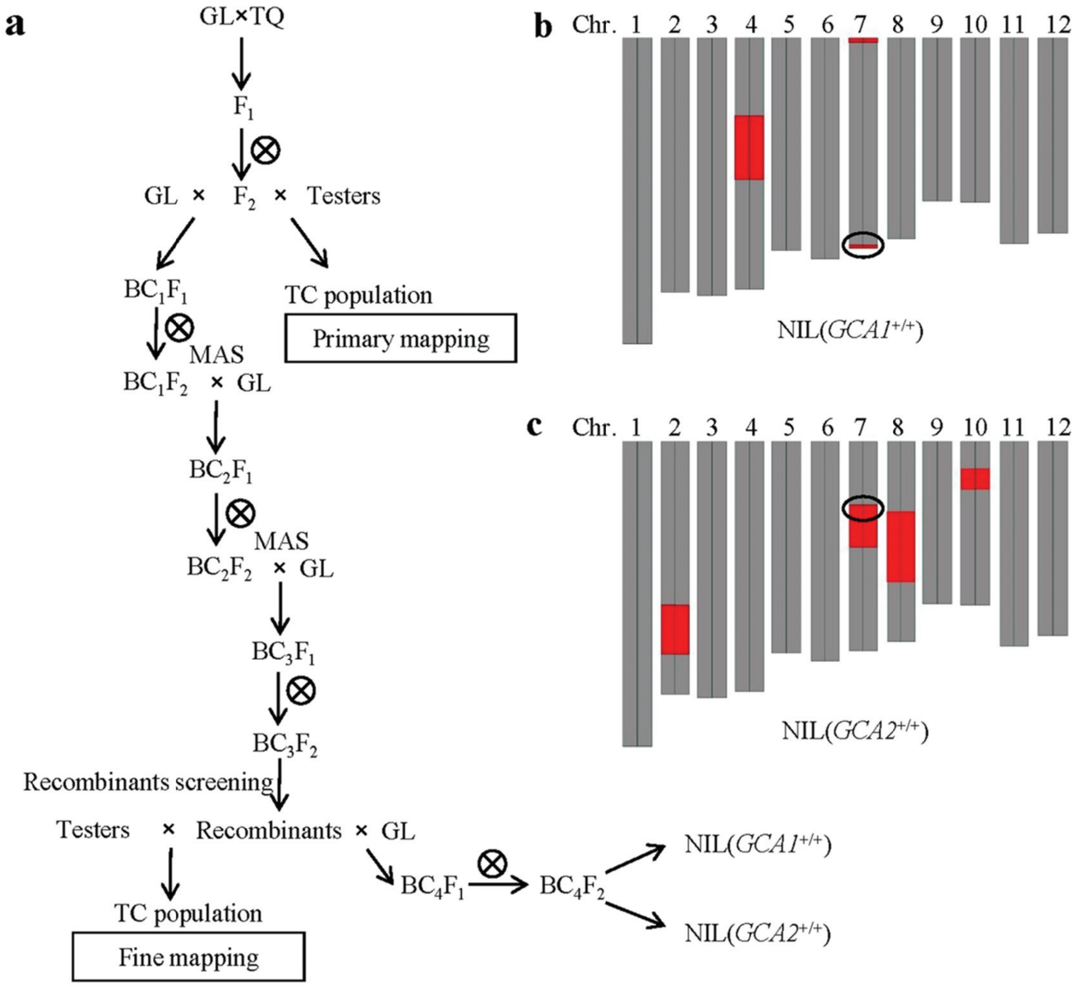

Supplementary Figure S2. Construction of populations for GCA QTL mapping and graphical genetic background for NIL(*GCA1*$^{+/+}$) and NIL(*GCA2*$^{+/+}$). (**a**) The TC population used for primary mapping was derived from crosses of the individual plants of F_2 population with five testers. The TC population used for fine mapping was derived from test crosses of the recombinant plant with five testers. TQ (donor) and GL (recurrent) were used as the parents. (**b, c**) Graphical genetic background for NIL(*GCA1*$^{+/+}$) and NIL(*GCA2*$^{+/+}$) determined by 139 genome-wide SSR and InDel markers. Gray and red bars indicate the GL chromosomes and TQ fragments, respectively. Black ovals indicate the positions of *GCA1* and *GCA2* in NIL(*GCA1*$^{+/+}$) and NIL(*GCA2*$^{+/+}$).

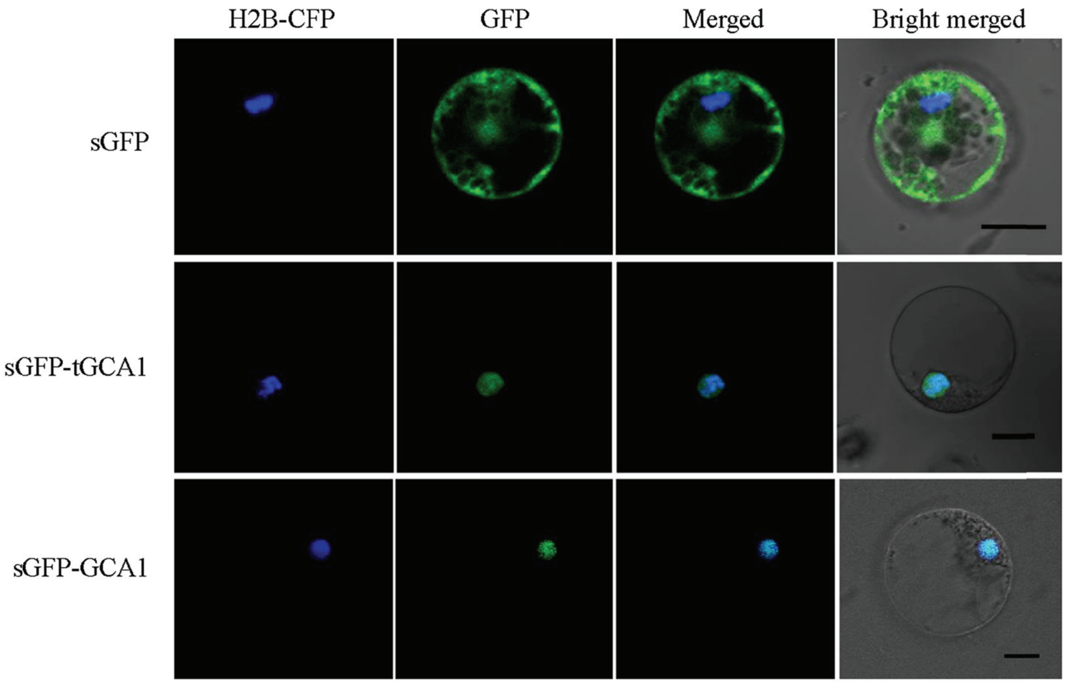

Supplementary Figure S3. Subcellular localization of full-length (GCA1) and truncated (tGCA1) GCA1 proteins. The influorescence microscopic images exhibiting that sGFP alone was observed throughout the cytoplasm, whereas, the sGFP-GCA1 and sGFP-tGCA1 fusion proteins were co-localized with the nuclear marker H2B-CFP in rice protoplasts. Scale bars =10 um.

Supplementary Tables

Supplementary Table S1: Estimates of GCA effects for DTH, PH and SPP in a half-diallelic cross analysis with six rice elite varieties

	YB	ZS	AJ	GL	L6	TQ
DTH	1.8	-6.9	-6.4	-6.9	15.4	3.0
PH	1.1	-7.8	-6.4	-10.5	16.2	7.4
SPP	53.7	-30.4	-36.1	-38.0	4.2	46.6

Supplementary Table S2: Recombination events identified in the candidate region in the BC_3F_2-*GCA1* population and estimated GCA effects for DTH, PH and SPP

Recombinants	ID77	S76	ID710	RM22140	RM1306	S77	S726	S722	GCA-DTH	GCA-PH	GCA-SPP	progeny test
270	GL	GL	GL	GL	H	H	H	H	3.80	4.52	4.96	H
337	GL	GL	GL	GL	H	H	H	H	5.00	0.57	-1.44	H
367	H	H	H	GL	GL	GL	GL	GL	-8.40	-4.32	-4.18	GL
471	H	H	H	H	GL	GL	GL	GL	-8.01	-3.11	-6.17	GL
966	GL	GL	GL	H	H	H	H	H	1.21	-0.60	0.39	H
1351	GL	GL	GL	H	H	H	H	H	2.20	1.50	6.89	H
1450	GL	GL	H	H	TQ	TQ	TQ	TQ	19.24	9.71	11.44	TQ
1540	H	H	H	H	GL	GL	GL	GL	-8.59	-6.47	-9.91	GL
1598	H	GL	GL	GL	GL	GL	GL	GL	-7.55	-3.74	-1.92	GL
1615	GL	TQ	TQ	TQ	TQ	TQ	TQ	TQ	20.15	10.11	4.09	TQ
1679	H	H	H	H	GL	GL	GL	GL	-8.42	-3.58	-0.36	GL
1847	TQ	H	H	H	GL	GL	GL	GL	-4.19	-2.12	-4.58	GL
1854	GL	GL	GL	GL	H	H	H	H	1.41	0.00	-0.04	H
2087	H	H	H	GL	H	GL	GL	GL	-9.30	-4.57	-5.29	GL
2108	GL	GL	GL	H	H	H	H	H	2.54	0.33	1.23	H
2373	H	H	H	GL	GL	GL	GL	GL	-7.00	-4.35	-6.17	GL
2462	H	H	H	GL	GL	GL	GL	GL	-7.85	-5.10	-7.85	GL
2554	GL	GL	GL	H	H	H	H	H	2.45	2.66	2.82	H
2718	GL	GL	GL	GL	H	H	H	H	4.79	0.10	4.43	H
2858	H	H	H	H	GL	GL	GL	GL	-8.30	-3.16	-1.31	GL

2941	GL	GL	GL	GL	H	H	TQ	TQ	18.57	10.08	14.29	TQ
3034	TQ	H	H	H	H	H	H	H	3.20	4.55	1.28	H
3092	H	H	H	H	GL	GL	GL	GL	-6.95	-3.04	-2.59	GL

Three genotype classes for respective markers and the *GCA1* locus: GL: homozygous for Guangluai #4; TQ: homozygous for Teqing; H: heterozygous. DTH, PH and SPP represent the corresponding GCA values estimated from the whole TC progenies of these recombinants. The progeny test was conducted based on the segregation conditions of the traits in the BC_3F_3 families and TC progenies.

Supplementary Table S3: Gene expression levels from RNA-seq data of GL and TQ in the candidate genomic region

Locus Name	GL-RPKM	TQ-RPKM	log2 Ratio(TQ/GL)	FDR	Gene Product Name
LOC_Os07g48890	2.393	1.888	-0.342	0.1836	expressed protein
LOC_Os07g48900	un	un	un	un	expressed protein\BHLH protein
LOC_Os07g48910	0.382	0.847	1.150	0.2125	retrotransposon protein, putative, unclassified, expressed
LOC_Os07g48920	4.723	6.705	0.505	1.39E-04	aldehyde dehydrogenase, putative, expressed
LOC_Os07g48930	un	un	un	un	expressed protein
LOC_Os07g48940	un	un	un	un	OsFBDUF39 - F-box and DUF domain containing protein, expressed
LOC_Os07g48950	0.981	1.209	0.302	0.5764	aldehyde dehydrogenase 22A1 precursor, putative, expressed
LOC_Os07g48960	2.334	6.143	1.396	6.95E-32	OsLonP3 - Putative Lon protease homologue, expressed
LOC_Os07g48970	20.413	31.628	0.632	2.86E-24	OsPOP17 - Putative Prolyl Oligopeptidase homologue, expressed
LOC_Os07g48980	3.273	0.483	-2.760	4.07E-07	nicotianamine synthase, putative, expressed
LOC_Os07g49000	0.862	0.242	-1.835	0.0147	DNAJ heat shock N-terminal domain-containing protein, putative
LOC_Os07g49010	11.787	11.255	-0.067	0.4882	TOPBP1B - Similar to DNA replication protein TOPBP1 from, expressed
LOC_Os07g49020	5.361	3.933	-0.447	0.5781	conserved hypothetical protein
LOC_Os07g49030	18.489	29.559	0.677	3.21E-19	PHD-finger family protein, expressed
LOC_Os07g49040	1.143	2.248	0.976	3.67E-05	protein phosphotase protein, putative, expressed
LOC_Os07g49050	0.565	1.139	1.013	0.3011	hypothetical protein/Conserved gene of unknown function
LOC_Os07g49060	un	un	un	un	retrotransposon protein, putative, unclassified
LOC_Os07g49070	7.484	11.227	0.585	6.43E-07	expressed protein
LOC_Os07g49080	13.983	29.760	1.090	7.41E-24	COBRA-like protein 7 precursor, putative, expressed
LOC_Os07g49090	4.409	10.042	1.188	2.08E-04	WD-40 repeat family protein, putative, expressed
LOC_Os07g49100	6.149	4.684	-0.393	0.1267	pectinesterase, putative, expressed
LOC_Os07g49110	58.004	78.036	0.428	2.25E-47	D-alanine--D-alanine ligase family, putative, expressed

Supplementary Table S3 (Continued)

Locus Name	GL-RPKM	TQ-RPKM	log2 Ratio(TQ/GL)	FDR	Gene Product Name
LOC_Os07g49114	19.734	6.137	-1.685	7.13E-12	wound-induced protein WI12, putative, expressed
LOC_Os07g49120	un	un	un	un	sex determination protein tasselseed-2, putative, expressed
LOC_Os07g49130	un	un	un	un	expressed protein
LOC_Os07g49140	7.522	4.571	-0.718	4.17E-06	expressed protein
LOC_Os07g49150	8.543	17.853	1.063	8.83E-26	26S protease regulatory subunit 4, putative, expressed
LOC_Os07g49160	0.090	0.001	-6.493	0.3419	retrotransposon protein, putative, unclassified, expressed
LOC_Os07g49170	un	un	un	un	retrotransposon protein, putative, unclassified, expressed
LOC_Os07g49180	0.501	0.276	-0.862	0.3544	retrotransposon protein, putative, unclassified, expressed
LOC_Os07g49190	un	un	un	un	retrotransposon protein, putative, unclassified
LOC_Os07g49200	0.782	0.394	-0.987	0.4453	membrane associated DUF588 domain containing protein, putative, expressed
LOC_Os07g49210	2.029	3.140	0.630	3.85E-05	helicase conserved C-terminal domain containing protein, expressed
LOC_Os07g49220	0.057	0.001	-5.825	0.5712	expressed protein
LOC_Os07g49230	1.672	5.891	1.817	3.51E-21	ubiquitin-activating enzyme, putative, expressed
LOC_Os07g49240	12.263	4.454	-1.461	2.80E-19	MRH1, putative, expressed
LOC_Os07g49250	un	un	un	un	thiamine pyrophosphate enzyme, C-terminal TPP binding domain protein
LOC_Os07g49260	4.167	4.979	0.257	0.0623	importin subunit beta, putative, expressed
LOC_Os07g49270	7.600	10.532	0.471	1.66E-07	AMP deaminase, putative, expressed
LOC_Os07g49280	5.249	15.886	1.598	1.45E-45	PMR5, putative, expressed
LOC_Os07g49290	un	un	un	un	PHD finger family protein, putative, expressed
LOC_Os07g49300	23.442	25.900	0.144	0.0111	expressed protein
LOC_Os07g49310	9.841	6.994	-0.493	0.0025	omega-3 fatty acid desaturase, chloroplast precursor, putative, expressed
LOC_Os07g49320	9.687	8.872	-0.127	0.1310	HEAT repeat family protein, putative, expressed

Supplementary Table S3 (Continued)

Locus Name	GL-RPKM	TQ-RPKM	log2 Ratio(TQ/GL)	FDR	Gene Product Name
LOC_Os07g49330	53.130	48.107	-0.143	0.0281	phospholipase C, putative, expressed
LOC_Os07g49340	un	un	un	un	transposon protein, putative, Mariner sub-class, expressed
LOC_Os07g49350	un	un	un	un	expressed protein
LOC_Os07g49360	1.439	0.484	-1.572	0.0523	peroxidase precursor, putative, expressed
LOC_Os07g49370	0.221	0.111	-0.987	0.3175	glycosyltransferase family 43 protein, putative, expressed
LOC_Os07g49380	27.082	27.407	0.017	0.8664	PWWP domain containing protein, expressed
LOC_Os07g49390	41.878	30.650	-0.450	9.37E-11	P-protein, putative, expressed
LOC_Os07g49400	86.375	108.185	0.325	2.53E-12	OsAPx2 - Cytosolic Ascorbate Peroxidase encoding gene 4,5,6,8, expressed
LOC_Os07g49410	11.964	12.975	0.117	0.3394	uncharacterized ACR, YagE family COG1723 containing protein, expressed
LOC_Os07g49420	un	un	un	un	retrotransposon protein, putative, unclassified, expressed
LOC_Os07g49430	0.023	0.184	3.013	0.0396	retrotransposon protein, putative, unclassified, expressed
LOC_Os07g49440	0.036	0.109	1.598	0.4671	retrotransposon protein, putative, unclassified, expressed
LOC_Os07g49460	143.731	47.583	-1.595	0.00E+00	response regulator receiver domain containing protein, expressed
LOC_Os07g49470	12.708	7.267	-0.806	2.98E-08	protein kinase APK1B, chloroplast precursor, putative, expressed
LOC_Os07g49480	24.941	22.523	-0.147	0.0617	(Kinase interacting protein 1)KIP1, putative, expressed
LOC_Os07g49490	un	un	un	un	retrotransposon protein, putative, unclassified, expressed
LOC_Os07g49500	0.023	0.023	0.013	0.9942	retrotransposon protein, putative, unclassified, expressed
LOC_Os07g49510	8.506	11.374	0.419	0.0031	expressed protein;
LOC_Os07g49520	2.579	8.921	1.790	1.57E-35	2-oxoglutarate dehydrogenase E1 component, mitochondrial precursor
LOC_Os07g49530	3.966	7.512	0.922	1.82E-06	MYB family transcription factor, putative, expressed
LOC_Os07g49540	un	un	un	un	expressed protein

Supplementary Table S4: Trait performance of NILs and *GCA1*^{OX} transgene-negative segregates (Neg), transgene-positive homozygotes (Pos)

Genotype	DTH	PH(cm)	SPP
NIL(*GCA1*$^{-/-}$)	70.9±0.8	85.9±1.9	153.4±12.3
NIL(*GCA1*$^{+/+}$)	90.2±0.7	108.5±2.2	215.3±10.4
P-value	6.54E-44	1.32E-30	7.39E-20
GCA1^{OX}-5(Neg)	71.1±1.4	80.7±3.8	119.9±8.5
GCA1^{OX}-5(Pos)	103.6±1.3	104.6±3.0	182.5±9.8
P-value	2.41E-42	1.51E-23	3.47E-23
GCA1^{OX}-9(Neg)	69.0±1.4	81.6±2.1	121.3±8.9
GCA1^{OX}-9(Pos)	104.5±1.8	108.9±2.5	185.7±8.7
P-value	4.59E-42	6.62E-32	3.03E-24

Means ± standard deviations were obtained from 20 plants for each genotype cultivated at Wuhan in the summer of 2014. The Student's *t*-test was used to reveal any significant differences in the agronomic traits between the different genotypes within NILs and *GCA1*^{OX} transgenic lines. The *P*-value was calculated with one-tailed *t*-test between the genotypes.

Supplementary Table S5: Molecular markers used in the fine mapping of *GCA1*

Marker name	Forward primer (5'-3')	Reverse primer (5'-3')
RM22140	GCAAAGCCAGGAGGTCTCTACC	TCAAGAAACCGAAACACGAAGC
RM1306	AGTCTCAAAGGCATACAGTACACACC	GCCAATTACCTTCCCGTACATAGC
ID77	GGAATGGTAGGAACCGGATTA	AAGCGACAAAGCCCAACGA
ID710	TGTGGGGTTAGGTTAGGAGCT	GGAATCTTGCATGTTGGTGAG
S76	CTGCTGGATTCAGTGGTCGTAT	TCAAAGATGGGCTCTTTCTGTTC
S77	CCCGCGATTAAGGAACGAAACC	CGGAAGCAGGAGCAAGAAAGCAT
S726	CGACAAGATCGTAATCGAAAGAT	GGAGCAGGCAACAGTAGACAC
S722	ACTTATTACCGTTGGTGGATG	AAAGAAGATGCCTCAGGAGTT

The SSR marker information was obtained from the Gramene website (http://www.gramene.org/). The indel and SNP marker information was obtained from previous genome sequencing data.

Supplementary Table S6: Primers used in this study

Primer name	Forward primer (5'-3')
371F	CGGGATCCATGATGGGAACCGCTC
371R	AAAACTGCAGAGGTCATCTGTCCGCTG
372R	AAAACTGCAGCTAGGCTTTGTCATTTGTAGTGGAACCCATG
q37.1F	CCACTACAAAGAACGTTGTGACAAA
q37.1R	CCATTAGCCTTAACAGCTGAGGGT
qActF	TGTATGCCAGTGGTCGTACCA
qActR	CCAGCAAGGTCGAGACGAA
Hom1F	AATCCTGTTGCCGGTCTTG
Hom1R	ATGTATAATTGCGGGACTCTAATC
RBE4F	GTTTTAGTTGGGTGAAAGCGGTT
RBE4R	CCTGTTAGTTCTTCCAATGCCCTTA

Primers 371F/371R and 371F/372R were designed for the amplification of full-length and truncated CDSs of GCA1, respectively. Primers q37.1F/q37.1R were designed for mRNA quantification of the TQ allele of *GCA1*, and qActF/qActR were used as an internal control. Primers Hom1F/Hom1R were used to identify the homozygous transgenic lines, and RBE4F/RBE4R were used as an internal control.
